# Comparison of Ultrasound and Scintigraphy in Gastric Emptying: A Comprehensive Review

**DOI:** 10.3390/diagnostics16172816

**Published:** 2026-09-01

**Authors:** Emilie Lein, Karen L. Jones, Odd Helge Gilja

**Affiliations:** 1 Department of Clinical Medicine, University of Bergen, 5007 Bergen, Norway; 2 School of Medicine, Adelaide University, Adelaide 5005, Australia; karen.jones@adelaide.edu.au; 3 School of Allied Health and Human Performance, Adelaide University, Adelaide 5005, Australia; 4 Endocrine and Metabolic Unit, Royal Adelaide Hospital, Adelaide 5000, Australia; 5 National Centre for Ultrasound in Gastroenterology, Department of Medicine, Haukeland University Hospital, 5021 Bergen, Norway

**Keywords:** gastric emptying, scintigraphy, ultrasound, stomach, gastric function

## Abstract

Gastric emptying is the process by which gastric contents are transferred from the stomach to the small intestine for further digestion and nutrient absorption. It is the result of multiple gastrointestinal mechanisms and is essential for understanding the kinetics of digestion in humans. Delayed gastric emptying is clinically relevant as it is commonly observed in conditions such as gastroparesis and functional dyspepsia. A wide range of diagnostic modalities are available to assess gastric emptying, including scintigraphy, ultrasonography, magnetic resonance imaging, gastric aspiration, and stable isotope breath tests. Scintigraphy is widely regarded as the ‘gold standard’ technique, as it enables a direct quantitative assessment of gastric emptying following ingestion of a radiolabeled meal(s). Scintigraphy is non-invasive, reproducible, widely available, and well tolerated; however, it lacks standardized protocols, is time-consuming, costly, and necessitates exposure to ionizing radiation. Although the radiation dose is low, cumulative exposure remains a concern, particularly in pediatric and pregnant populations. In contrast, ultrasonography is safe, non-invasive, portable, inexpensive, and free from radiation exposure. Nevertheless, the application of ultrasound is limited by operator dependency and the need for technical expertise, and image quality may be affected by factors such as obesity and intraluminal gas. Furthermore, there is substantial methodological variability across studies, and its use in assessing gastric emptying of semisolid and solid meals is less well established. 3D ultrasonography, in particular, shows promise as a safe, non-invasive method for assessing gastric emptying. Further studies are required to promote the consistent application of established scintigraphic gastric emptying protocols across centers and to validate 3D ultrasound, particularly with solid meals, to potentially enhance its role as an alternative to scintigraphy. This article represents a comprehensive review based on a structured literature search with the primary aim of comparing ultrasonographic and scintigraphic measurements of gastric emptying, including the advantages and disadvantages of both methods.

## 1. Introduction

Gastric emptying refers to a complex process by which the stomach stores, grinds and transfers its contents into the duodenum for further digestion and nutrient absorption [1]. It is a major area of research for understanding the kinetics of food and liquid digestion and absorption. Furthermore, it has clinical relevance in conditions such as functional dyspepsia [2,3,4] and gastroparesis [5]. The stomach performs several coordinated functions, including gastric accommodation, generation of a pressure gradient between the proximal and distal stomach, grinding and sieving of food [6,7], and antro-pyloro-duodenal contractility and coordination [8]. Gastric emptying of both solids and liquids results from the interaction of these mechanisms and is considered one of the most important physiological functions of the stomach [8].

A major focus of recent research relates to glucagon-like peptide-1 (GLP-1) receptor agonists, which are widely and increasingly used to treat type 2 diabetes mellitus and obesity [9]. One of the important mechanisms of their action is slowing of gastric emptying [9,10]. This has stimulated renewed interest in methods to estimate gastric emptying, further supported by the development of modern 3D and matrix-transducer techniques, and point-of-care gastric ultrasound [11,12].

Numerous methods have been proposed to evaluate gastric emptying, including scintigraphy, ultrasonography, magnetic resonance imaging (MRI), gastric aspiration, the paracetamol absorption test, and stable isotope breath tests [13,14,15]. Scintigraphy is currently regarded as the clinical “gold standard” technique for measurement of gastric emptying; however, it has several limitations. While it provides a direct and non-invasive quantification of gastric emptying, there is a lack of standardization with respect to test meal composition, patient positioning, timing of image acquisition, and availability of appropriate normal reference values. In addition, scintigraphy requires expensive equipment that is not always readily available, necessitates exposure to ionizing radiation, and is time-consuming [8]. Two-dimensional (2D) ultrasonography was first compared with scintigraphy in 1986 by Holt et al. [16,17], and later by other authors [3,18,19,20,21,22,23]. These studies found close concordance between gastric emptying assessed by 2D ultrasound and scintigraphy, simplifying the method by limiting the measurements to a single scan of the antrum. Hveem et al. [24] reported a close correlation between scintigraphic measurements of total stomach emptying of both nutrient and low-nutrient liquids and changes in antral area. More recently, 3D ultrasonographic techniques have been developed to measure gastric emptying [20,25], including matrix-transducer techniques [26] and magneto-based ultrasonography [27]. Both 2D and 3D ultrasonography can be used to assess gastric emptying, while providing additional information on gastric function [28]. Accordingly, ultrasonography can assess accommodation [29,30], intragastric distribution [25], antral contractility and distensibility [31,32,33,34], and transpyloric flow [35,36]. Moreover, the strain of the gastric antrum can be assessed non-invasively by ultrasound techniques [32,37]. There are some limitations, particularly impaired visualization due to intraluminal gas or obesity. Furthermore, ultrasonography is relatively operator-dependent and is generally considered more reliable for assessing gastric emptying of liquids than solids [1]. To our knowledge, no review has specifically focused on the comparison of gastric emptying measurements obtained by ultrasonography and scintigraphy, while critically examining the strengths and limitations of both methods. In this comprehensive review based on a structured literature search, the aims were to compare gastric emptying measurements obtained by ultrasonography and scintigraphy and to consider the advantages and disadvantages of both methods.

## 2. Materials and Methods

This is a comprehensive review based on a structured literature search that was conducted in PubMed and Embase in December 2024. The search was initially limited to studies published between 1984 and 2024 in PubMed and 1972–2024 in Embase. The search strategy combined controlled vocabulary and free-text terms related to gastric emptying, ultrasonography and scintigraphy, using Boolean operators (AND/OR). The search strategy was revised twice to improve specificity and reduce irrelevant records. First, alternative ultrasound-related search terms were evaluated and incorporated. Second, the term “human” was added to limit the search to studies conducted in human subjects. The following keywords were used: gastric emptying, ultraso*, scintigraphy, and human. The PubMed search yielded a total of 80 articles, whereas the Embase search yielded 86 articles. Of the Embase articles, 54 articles overlapped with the PubMed search and 4 articles were duplicated within Embase, leaving 28 additional articles for screening. Duplicated articles were excluded before title and abstract screening. The finalized search is summarized in Appendix A. Two reviewers independently evaluated the articles, with disagreements resolved through discussion and consensus. The study was not designed as a systematic review, and no formal risk-of-bias or certainty-of-evidence assessment was performed. In total, 108 articles underwent a two-stage screening process. The first phase consisted of screening titles and abstracts and included articles where both ultrasound and scintigraphy were addressed or applied to validate methods related to gastric emptying. Specific inclusion and exclusion criteria were applied during the screening process. Studies were included if they involved humans, assessed the stomach, and used ultrasonography and/or scintigraphy as the primary method of investigation. Eligible outcomes focused on the evaluation or comparison of ultrasound with scintigraphy in relation to gastric emptying. Only articles published in English, French, or German were included. Consequently, relevant studies published in other languages, such as Japanese and Croatian, may have been excluded. Furthermore, studies were excluded if they involved animals, focused on organs other than the stomach (e.g., the gallbladder), or employed alternative assessment methods such as breath tests or single photon emission computed tomography (SPECT). Moreover, studies reporting outcomes unrelated to gastric emptying, such as transpyloric flow, therapeutic interventions, or botulinum toxin injections, were excluded.

**Figure 1:**
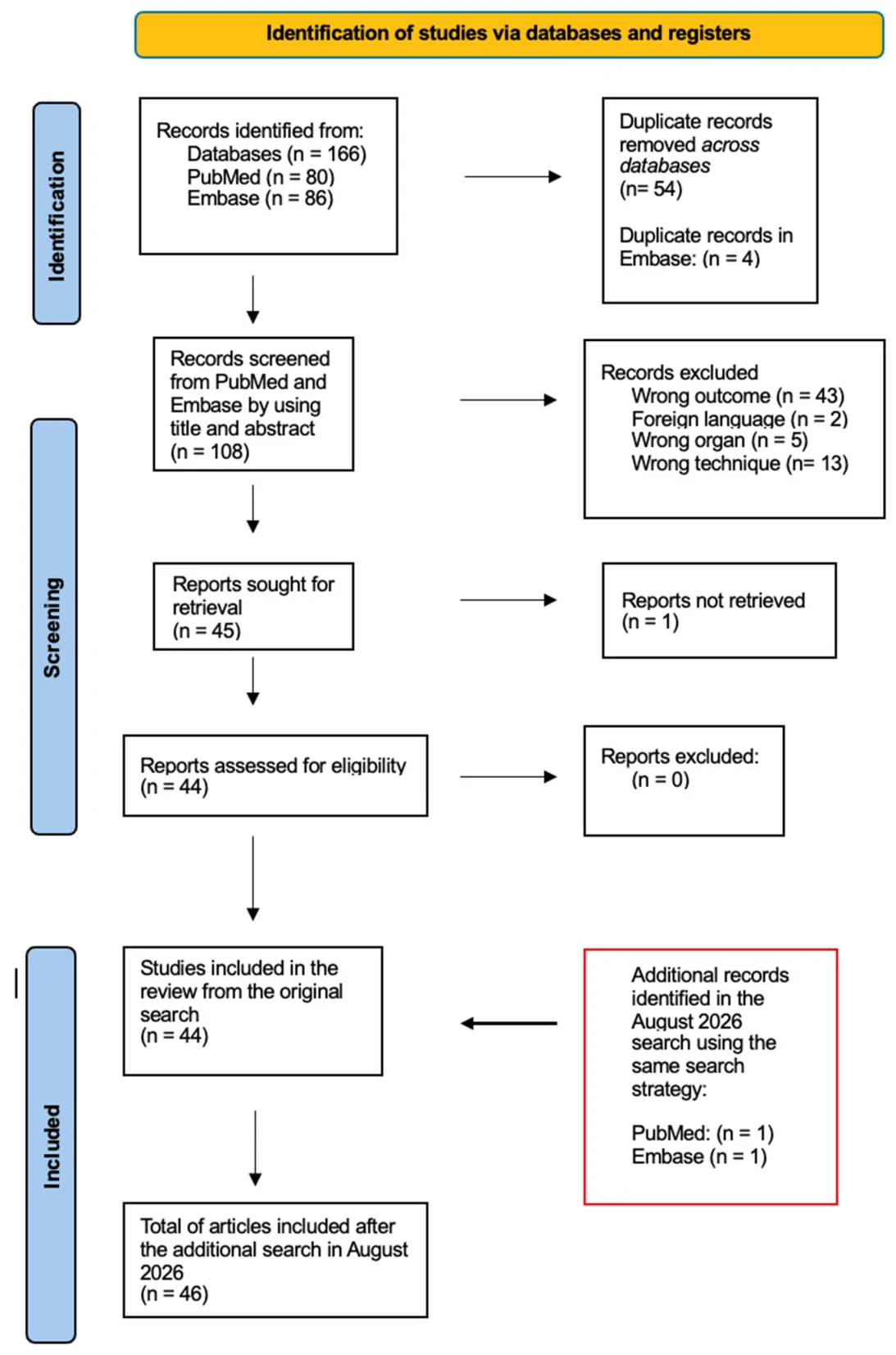
PRISMA diagram of studies of ultrasound and scintigraphic assessment of gastric emptying.

The second phase consisted of reading the articles in full text. One Korean article was excluded as the correct full text could not be retrieved. Initially, 44 articles were selected for inclusion in this study. An additional literature search using the same search strategy was conducted in August 2026 and identified 2 articles relevant to the present review that were not included in the original search [38,39]. One article identified through PubMed directly compared ultrasonography with scintigraphy [38], whereas one additional article identified through Embase was a narrative review published in English that provided relevant information on both methods [39]. In total, 46 articles were included in this review. A thorough overview of the screening process is shown in the PRISMA diagram (Figure 1). For each article, we extracted the year of publication, the purpose of the study, the protocol, meal composition and caloric content, symptom scoring and symptoms recorded, subject numbers, relevant characteristics of the participants, and the main conclusion relating to the technique used.

## 3. Results

Of the 46 included studies, 19 articles compared gastric emptying measured by 2D ultrasound with scintigraphy, while 3 articles compared 3D ultrasound to scintigraphy, and these are presented in Table 1. Table 2 summarizes the advantages and disadvantages of ultrasound and scintigraphy. Among the included studies, 13 studies reported a good correlation between ultrasound and scintigraphy when measuring the gastric half-emptying times [3,16,18,20,21,38,40,41,42,43,44,45,46], while 3 studies showed good correlation or concordance between scintigraphy and ultrasound when total gastric emptying times were assessed [3,19,24] and 2 studies showed a good correlation between ultrasound antral volume/area and scintigraphy [47,48].

### 3.1. Scintigraphy

#### 3.1.1. Method/Principle/Procedure

**Figure 2:**
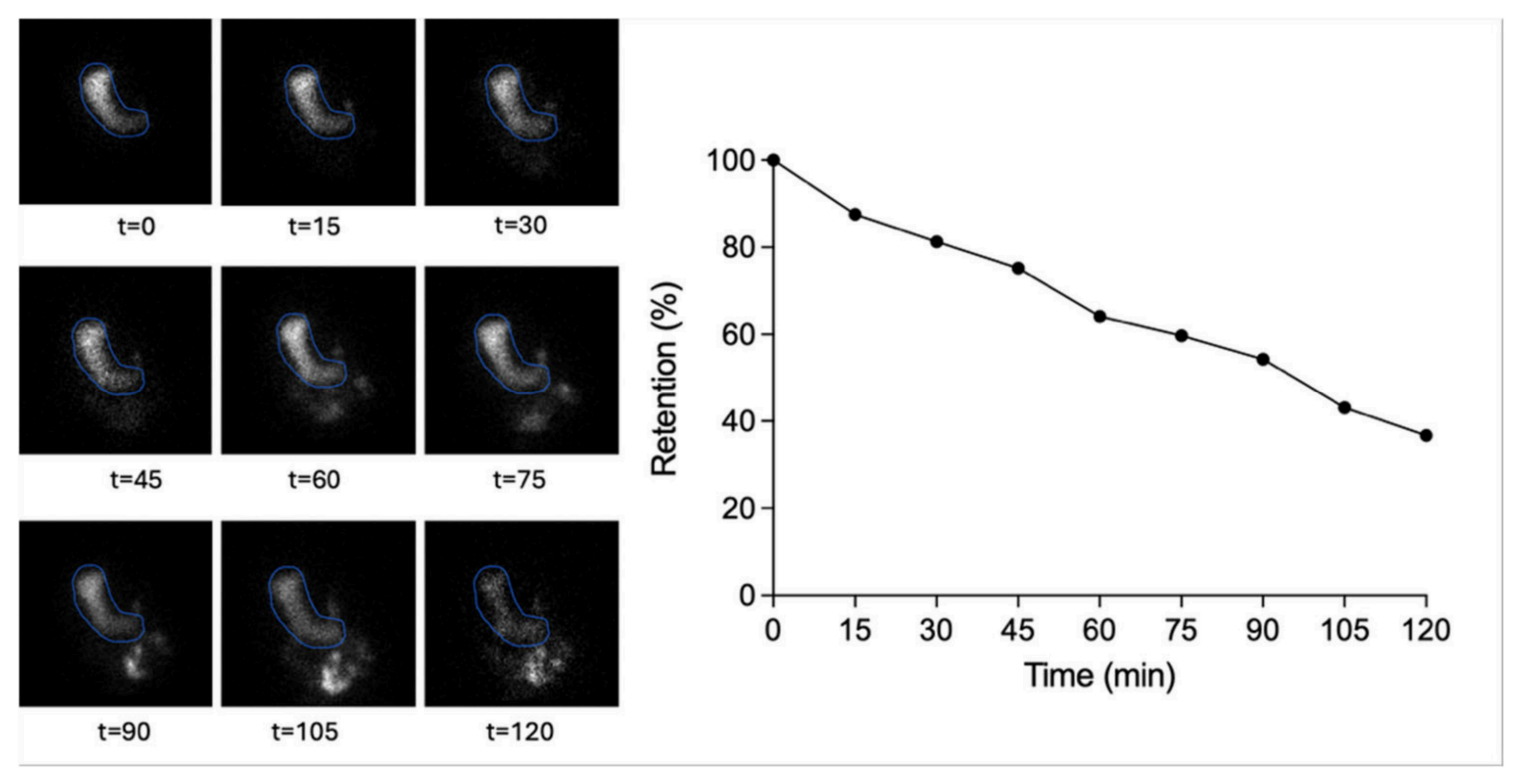
A figure demonstrating posterior scintigraphic images in a healthy individual who ingested a test drink of 75 g glucose in 300 mL radiolabeled with ^99m^Tc-DTPA. Data were corrected for radionuclide decay, subject movement and tissue attenuation using factors derived from a left lateral image. Gastric emptying is linear and expressed as % retention in the stomach over time.

Measurement of gastric emptying with scintigraphy is commonly performed using a gamma camera [17]. The patient fasts for at least 6 h and ingests a standardized test meal labeled with a radioactive isotope [53]. An advantage of this technique is that emptying of both solid and liquid components can be measured simultaneously using two radioisotopes. The transit of the radiolabeled meal through the stomach and into the small intestine can then be monitored almost continuously. Regions of interest (ROIs) are drawn around the gastric area, and by employing either a dual-head camera system, alternating anterior and posterior abdominal scans, or posterior scans with a single lateral image, corrections can be made for tissue attenuation and photon scatter, as well as radionuclide decay, to improve the accuracy and reliability of the measurements [53,54]. Gastric emptying parameters, such as the percentage of gastric retention at predefined time points after ingestion and the gastric half-emptying time (T50), defined as the time required for 50% of the radioactive tracer to be emptied from the stomach, are obtained and compared with an established normal range, under the assumption that the isotope empties in parallel with the test meal [17,55]. This is illustrated in Figure 2, which presents a representative example of scintigraphic assessment and demonstrates how gastric emptying measurements are visualized. The lag time (Tlag), defined as the time required for the test meal to first appear in the small intestine, is also frequently reported. These variables are calculated depending on the image acquisition methods and the mathematical modeling assumptions applied to measure gastric emptying [55].

##### Test Meals

The composition of test meals differs considerably between studies, which limits direct comparisons of gastric emptying results. Meal composition ranges from liquid to solid meals with either high- or low-nutritional content. Differences in the characteristics of meals influence gastric emptying through distinct physiological patterns [1]. Gastric emptying of solid meals is typically characterized by an initial lag phase reflecting the time taken for solid food to redistribute from the fundus to the antrum and for the antrum to grind food into particles smaller than 1–2 mm prior to emptying, followed by an overall linear emptying pattern [1,20], with an emptying rate of approximately 1–3 kcal per minute in health. Similarly, high-nutrient liquids empty in a linear fashion, after a very short lag phase [20]. This regulated emptying reflects inhibitory small intestinal feedback mechanisms mediated by nutrient-sensitive receptors located in the duodenum, which act to slow gastric emptying in response to nutrient load [1,20]. In contrast, non-nutrient or low-nutrient liquid meals empty more rapidly and follow a mono-exponential, volume-dependent emptying pattern, as they do not sufficiently stimulate the feedback mechanisms that regulate gastric emptying [1,20].

These differences in emptying patterns were demonstrated in a study by Gentilcore et al. [20] where ten healthy volunteers were studied with scintigraphy and a three-dimensional (3D) ultrasonography method. Participants ingested a liquid test meal in the form of either a high-nutrient dextrose solution (300 mL water containing 75 g dextrose; 314 kcal) or a low-nutrient beef soup (12 kcal). The study found that scintigraphic measurements of gastric emptying revealed a short lag phase for both test meals (soup: 2.8 ± 0.9 min vs. dextrose: 3.0 ± 1.1 min; *p* = 0.67). Following the lag phase, gastric emptying of the low-nutrient soup approximated a mono-exponential, whereas the high-nutrient dextrose solution followed an overall linear emptying pattern. Gastric emptying of the soup was markedly faster than that of the dextrose drink, as reflected by the scintigraphic half-emptying time (ScT50: soup 27.7 ± 4.8 min vs. dextrose 122.2 ± 13.3 min; *p* = 0.005). These findings attest to the importance of meal composition when interpreting and comparing gastric emptying results.

Meal composition was heterogeneous across the included studies and is summarized in Table 1, which provides an overview of the studies comparing gastric emptying assessed by scintigraphy and ultrasonography. Liquid test meals comprised a range of preparations, including meat soup, milk, dextrose drinks, water-based solutions, nutritional supplements, saline solutions, and orange juice. Solid meals varied from mixed full meals to standardized components such as chicken liver, pasta, eggs, and pancakes. Semisolid meals included preparations such as rice pudding, cellulose-based pastes, and mixtures containing ultrasonic contrast agents. The heterogeneity of meal compositions across the included studies limits the ability to make direct comparisons of gastric emptying measurements by scintigraphy.

In addition to variations in meal composition, the radiopharmaceuticals used for labeling differed substantially depending on the specific meal components. Commonly used radioisotopes include ^99m^Tc, ^111^In, and others, as summarized in Table 1. The choice of radiotracer is important and must satisfy several criteria. The tracer should be non-toxic, with radiation exposure maintained within accepted safety limits, and it must permit accurate quantification. Furthermore, it should not interfere with neurohumoral mechanisms regulating gastric emptying, such as gastric secretion and motility. The tracer must also be non-absorbable, remaining firmly bound to the test meal throughout the examination rather than being absorbed by the gastric mucosa or separating into another meal phase [56]. One of the first validated solid meals was an in vivo ^99m^Tc-sulfur colloid-labeled chicken liver prepared by supravital injection [56,57]. Although this technique produced stable labeling, it was impractical for routine clinical use [58]. Simpler approaches were later introduced, involving the mixing of 11–18 MBq (300–500 µCi) of ^99m^Tc-sulfur colloid with eggs or a fat-free egg substitute to create a standardized solid meal [58].

In 2008, the American Neurogastroenterology and Motility Society and the Society of Nuclear Medicine published a joint consensus report providing recommendations for gastric emptying scintigraphy, including a standardized solid-phase meal to improve comparability and test reliability [53]. The recommended meal consists of 120 g (60 kcal) liquid egg whites (EggBeaters^®^ or equivalent), corresponding to the volume of two large eggs, mixed with ^99m^Tc-sulfur colloid and cooked to a firm consistency. This is served with two slices of white bread (120 kcal), 30 g strawberry jam (75 kcal), and 120 mL of water. The total caloric content of the meal is 345 kcal, and the overall nutritional composition of the meal is approximately 72% carbohydrate, 24% protein, 2% fat, and 2% fiber [53,57]. Despite these recommendations, adherence to the standardized meal protocol remains relatively low, compromising direct comparisons between studies [42,59].

Importantly, standardized alternatives to the egg-based meal must be available, as some patients may have allergies, intolerances, or other dietary restrictions that limit or preclude their ability to consume certain meal components [57]. In addition, the test meal should be palatable to ensure that patients can comfortably consume the entire portion within the required time frame and be appealing to a broad patient population, including children [57]. The meal should also be suitable for use in comparative studies across different imaging techniques, allowing gastric emptying measurements obtained with scintigraphy to be compared with those from other diagnostic modalities. A number of alternative test meals have been investigated, including dry preparations such as oatmeal and pancakes, as well as more child-friendly options incorporating cheddar cheese. Liquid nutritional supplements, such as Ensure^®^, have also been evaluated. These meals are typically labeled with ^99m^Tc-sulfur colloid [57]. In addition, ^111^In-labeled radionuclides have been studied in both solid and liquid meal formulations, in which ^111^In-DTPA has been typically used in the liquid phase studies as it is chemically inert and does not bind to the solid phase of the meal, allowing concurrent assessment of both liquid and solid emptying [58]. However, these alternative approaches lack well-established reference values for gastric emptying based on large populations of healthy subjects [57].

At present, only ^99m^Tc-sulfur colloid is approved by the U.S. Food and Drug Administration for oral administration. Most other radiopharmaceuticals, including ^111^In-labeled compounds, are not approved for this indication and therefore must be specifically authorized and listed under the institution’s radioactive materials license [57].

##### Differences in the Technique

A number of additional methodological variations exist in scintigraphic protocols to assess gastric emptying. These include differences in the timing of image acquisition, the definition of the region of interest used to measure gastric retention, the projection angle of the gamma camera, and the positioning of the patient during the examination. These differences are summarized in Table 1. The timing and frequency of image acquisition vary considerably depending on the composition of the test meal. Some protocols involve rapid imaging over a 1 h period, whereas others extend up to 4 h. Current consensus recommendations advise obtaining images at a minimum of 0, 1, 2, and 4 h after meal ingestion, with additional time points acquired if clinically indicated [53].

The region of interest (ROI) is typically defined manually using an irregular ROI program to outline the stomach. The selected ROI may encompass the entire stomach and/or divide it into proximal and distal regions for more detailed analysis. It is important that the ROI includes both the fundus and the antrum while avoiding inclusion of the adjacent small bowel, which may lie in close proximity to the stomach [53].

Image acquisition can be performed using anterior, posterior, or simultaneous anterior and posterior projections, with the latter allowing calculation of the geometric mean. This approach is generally recommended because the movement of solid contents from the posteriorly located fundus to the anteriorly located antrum may artificially increase anterior counts due to reduced tissue attenuation, however other techniques for correction of tissue attenuation, such as acquiring left anterior oblique images [60] or using factors derived from a left lateral image of the stomach, following posterior imaging [61], have been used. Without correction, tissue attenuation can make gastric emptying appear slower than it is by introducing non-uniform attenuation [16,58].

The position of the subject can affect gastric emptying and therefore needs to be standardized during imaging [16]. By placing the individual in the seated position, there is an increased probability that, in some, a portion of the duodenum will be located posterior to the stomach compared to imaging in the supine position. This overlap of the small intestine can limit the accuracy of the scintigraphic measurements [16]. Measurements of gastric emptying in the sitting position, however, simulate a normal, physiological posture for eating, potentially resulting in more accurate measurements [21].

#### 3.1.2. Advantages

Scintigraphic measurements of gastric emptying are relatively easy to perform, widely available in urban areas and entail a reasonably low radiation burden [58]. The radiation burden is less than that of an X-ray and consequently is considered acceptable for single or sequential studies [62]. It allows accurate, quantitative measurement of gastric emptying, with minimal operator-related errors. By employing two different radiopharmaceuticals, it is possible to simultaneously assess both liquid and solid emptying as the gamma camera can detect two radioisotopes with different energies [4,58,62]. ^99m^Tc is inexpensive, widely available, and can be incorporated into liquid, solid and mixed solid meals [62]. Measurements of gastric emptying using solid meals are generally preferred over those using nutrient-containing liquids or semisolids, as abnormalities in emptying may be more frequently observed in symptomatic patients and more closely reflect food-induced symptoms. Furthermore, the impact of dilution may be greater with liquids compared to solids [13,17]. This is evident in patients with diabetic gastroparesis, in whom emptying of low-nutrient liquids is often normal, whereas delayed emptying is more commonly observed with solids and high-nutrient liquids [43]. Abnormalities in gastric emptying of liquids sometimes become apparent only in the presence of severe gastroparesis, although some studies have reported an increase in sensitivity in detecting gastroparesis when assessing gastric emptying of liquids, particularly when the latter contains nutrients [17,63].

Scintigraphy also demonstrates good diagnostic performance in motility disorders of the upper digestive tract [58]. The method can be used to assess impaired gastric emptying and antral motor function, as well as antral contraction patterns and intragastric meal distribution [13,43,62]. Occurrence of gastric contractions can be inferred from changes in isotope activity within small regions of interest [62]. Intragastric meal distribution is typically assessed by measuring changes in isotope activity within different regions of the stomach, commonly divided into proximal and distal segments [24,43,64]. Evaluation of intragastric meal distribution may be clinically relevant, as abnormalities are frequently observed in patients with diabetic gastroparesis and other gastric motility disorders, including functional dyspepsia and gastroesophageal reflux disease. In diabetes, impaired intragastric meal distribution may reflect disordered coordination between the antrum and the duodenum, increased pyloric contractions, and/or impaired relaxation of the proximal stomach [43].

#### 3.1.3. Disadvantages

Well-recognized limitations of scintigraphic assessment of gastric emptying include the lack of methodological standardization with regard to meal composition, patient positioning, timing of image acquisition and lack of appropriate normal values [8,14]. Variations in test meals, correction factors, analyses and interpretations of results compromise direct comparisons between studies [62].

Scintigraphy is also limited in its ability to measure gastric and salivary secretions [17,65]. Gastric secretions are produced by endocrine cells in the gastric wall in response to the presence of different macronutrients and consist of a unique mixture of satiety-related peptides [1]. These secretions contribute to the overall gastric volume and may account for up to 188 mL/h [17], having a potentially significant impact on gastric emptying as a result of dilution. However, the radionuclides used in scintigraphy measurements do not bind to gastric secretions and are, therefore, not included in the measurements [13]. The radioisotope may also dissociate from the test meal and empty with the liquid component of the meal, potentially resulting in falsely increased estimates of gastric emptying rates [13,55]. Therefore, the binding characteristics of the isotope to the substrate are important to consider when interpreting results [13].

Gastric emptying measurements may be compromised by overlap between the small intestine and the distal stomach. As a result, the radiolabeled meal may appear to remain within the stomach when it has in fact already passed into the duodenum [16,54]. Other limitations include the time-consuming nature of the procedure for both patients and personnel [13,17,55]. This is largely due to the requirement for repeated imaging over a period of 2–4 h, during which patients are typically required to remain seated between image acquisitions [17]. Some studies have been conducted over a shorter duration (<2 h), estimating gastric emptying by extrapolating half-time values or the proportion emptied at 4 h using power-exponential analysis. However, this approach may introduce erroneous assumptions and inaccuracies in the estimation of gastric emptying at 4 h [8].

It should be appreciated that scintigraphy cannot distinguish between the different layers of the gastric wall and repeated measurements increase exposure to ionizing radiation [16], which restricts its use in pregnant women and children [1,43]. The method is also relatively expensive [62]. Most gastric emptying studies commence imaging at the end of meal ingestion, which assumes that fasting gastric content is very small and that no emptying of the stomach occurs during meal ingestion [20]. However, Gentilcore et al. [20] and others have frequently demonstrated significant gastric volumes prior to meal ingestion.

#### 3.1.4. Clinical Applications

Scintigraphy is frequently used to assess gastric emptying in patients with dyspepsia [66] and a variety of other systemic diseases that may affect gastric emptying [58]. It is generally indicated in several conditions when morphological investigations fail to reveal the cause of dyspepsia, such as postprandial nausea, vomiting, upper abdominal discomfort, early satiety and severe reflux esophagitis unresponsive to medical treatment [58], although it should be recognized that the relationship between the rate of gastric emptying and the presence of gastrointestinal symptoms is relatively weak [62]. Studies have been performed involving various forms of gastroparesis—diabetes patients, progressive systemic sclerosis and post-surgical conditions [8]. Post-surgical gastroparesis is generally caused by damage to or entrapment of the vagus nerve, and this occurs most commonly with fundoplication or bariatric surgical interventions [67]. As vagal innervation plays an important role in gastric accommodation and coordinated antral and pyloric motor function, vagal injury may impair gastric motility and result in delayed gastric emptying. Scintigraphy can demonstrate this disturbance as prolonged retention of a radiolabeled meal within the stomach.

Furthermore, scintigraphy can be used to assess gastric motility prior to surgical interventions such as fundoplication, small bowel transplantation, or even colectomy. It may also be applied in the screening of patients living with diabetes for delayed gastric emptying, particularly when treatment with medications that may further delay gastric emptying is being considered. In addition, it can be used in the evaluation of patients presenting with diffuse symptoms of gastrointestinal motility disorders, often in combination with assessment of small bowel and colonic transit [8].

### 3.2. Ultrasound

#### 3.2.1. Method/Principle/Procedure

**Figure 3:**
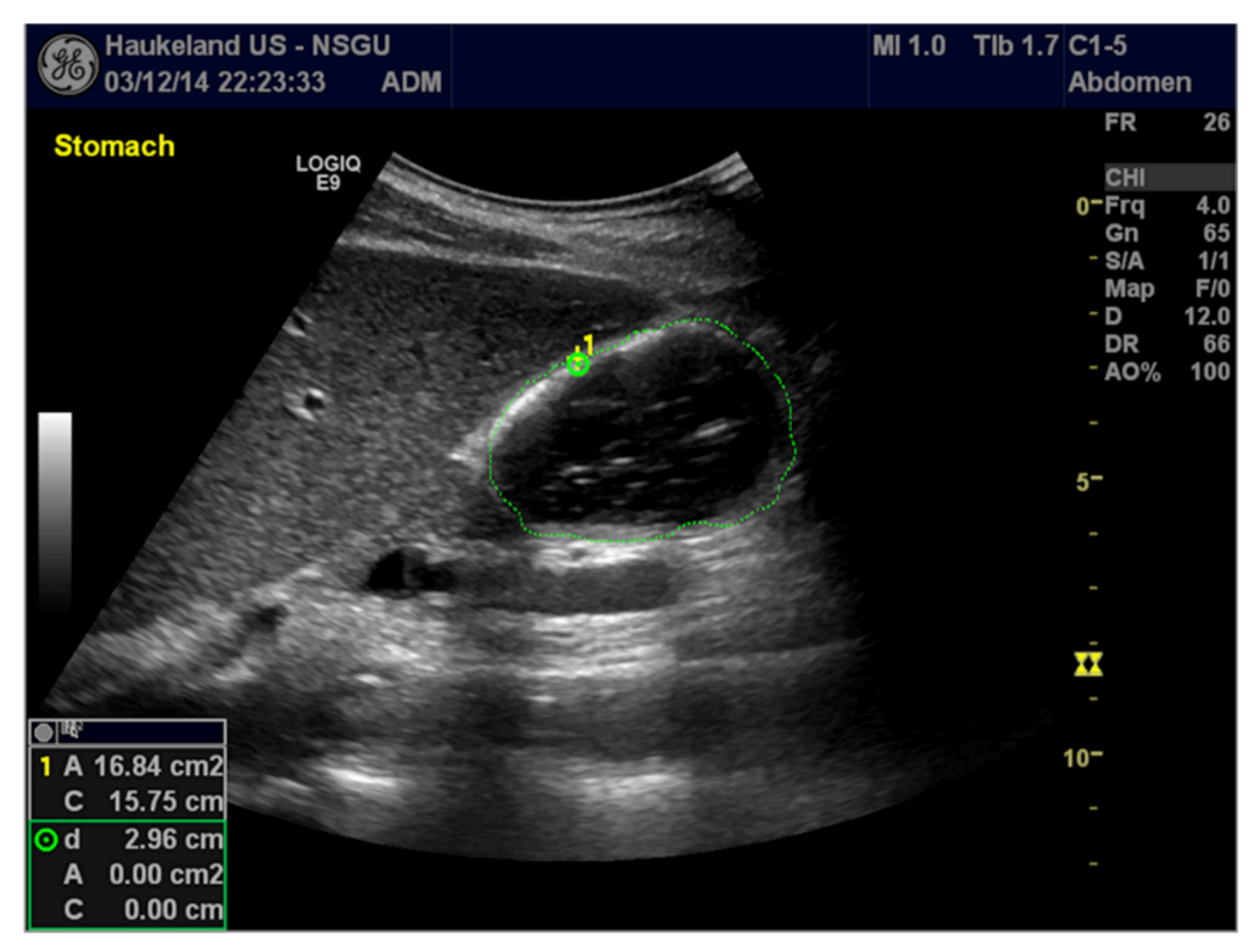
A 2D ultrasonographic image showing the gastric antrum area. The antrum is measured in the sagittal plane, using the aorta and superior mesenteric vein as anatomical landmarks. An increased gastric antrum area may be associated with dyspepsia.

Ultrasound is a non-invasive method that can be used to assess gastric emptying. The technique typically involves cross-sectional imaging of a defined region of the stomach, most often the antrum. Anatomical landmarks, commonly the superior mesenteric vein and aorta in the longitudinal axis, are applied to ensure standardization of the antral ultrasound sections. This view is illustrated in Figure 3. The patient is required to fast for at least 6 h, usually overnight, prior to the examination. They are placed in either a supine or seated position, after which a baseline scan is performed to assess residual gastric content. A standardized test meal is then administered, and repeated cross-sectional measurements are obtained at regular intervals, typically every 5–10 min over a period of 30–90 min, depending on the aims and protocol of the study. Accurate visualization of the stomach is essential to reliably calculate gastric volume from the cross-sectional images, which is derived using mathematical models or 3D acquisition of images. The half-emptying time is typically reported and defined as the time at which 50% of the gastric contents have emptied (T50), similar to scintigraphy [17].

Assessment of gastric emptying using ultrasonographic evaluation of the antrum can be performed using several different approaches. Methods include measurements of the antral volume [65], cross-sectional diameter [19], product of two diameters [18,41,46] and parasagittal or longitudinal area [2,3,24,47,48,49,50,68,69]. When antral area or antral diameter measurements are used to estimate gastric emptying, they are considered indirect measures of gastric contents, as emptying is inferred from changes in antral size relative to its postprandial maximum compared to its basal value and not the retention of content, implying that the meal has emptied from the stomach. In contrast, scintigraphy provides direct assessment of gastric emptying by quantifying the retention of intragastric contents. Hence, these two techniques measure different but related aspects of gastric emptying.

Bolondi et al. [70] introduced an ultrasonographic technique in 1985 based on antral volume measurements. Measurements derived from antral cross-sectional area, calculated using longitudinal and anteroposterior diameters, were compared with antral volume measurements obtained from the antero-pyloric region after ingestion of a solid 800 kcal meal in both healthy subjects and patients with dyspepsia. Longitudinal and anteroposterior diameters were measured at three standardized levels: the angular incisure, the pylorus, and an intermediate level corresponding to the scanning plane through the superior mesenteric vein, along with measurement of the antral length from the pylorus to the angular incisure. Antral volume was then calculated using a geometric model assuming elliptical cross-sections and linear variation in the axes along the antrum. When the two methods were compared, antral volume measurements were found to be more accurate than antral area measurements; however, the antral area method was also considered accurate and much simpler to perform.

Duan et al. [3] conducted a study similar to that of Bolondi et al. in patients with non-ulcer dyspepsia, comparing gastric emptying assessments obtained using Bolondi’s ultrasonographic antral volume method to scintigraphic measurements, in addition to antral area measurements derived from a sagittal cross-sectional view passing through the inferior vena cava in the knee- chest position. Total emptying time and half-emptying time were reported as outcome measures. Strong correlations were observed between scintigraphic measurements and assessment of both antral area (r = 0.9221) and antral volume (r = 0.9717), and between antral area and antral volume measurements (r = 0.9254), with all correlations reaching statistical significance (*p* < 0.0005). No significant differences were found in either final emptying time or half-emptying time between the ultrasonographic methods and scintigraphy. These findings suggest that ultrasonographic and scintigraphic measurements of gastric emptying are closely related, supporting the potential use of ultrasonography to assess gastric emptying. Consistent with the findings of Bolondi et al., the authors suggested that the antral area-based method was simpler to perform than the antral volume method.

Hveem et al. [24] compared ultrasonographic measurements of the antral cross-sectional area obtained from a single standardized longitudinal section, identified using the abdominal aorta and superior mesenteric vein as anatomical landmarks, with scintigraphic measurements of gastric emptying in seven healthy subjects after ingestion of both high- and low-nutrient liquid meals at various time points after ingestion. Antral area was measured using calipers and a built-in calculation program and expressed as a percentage of the maximal postprandial area at successive time points. These values were plotted against time to determine the half-emptying time (T50), with results presented as mean ± SEM. Comparison of T50 values obtained by ultrasonography and scintigraphy demonstrated a close correlation between the two methods, and the limit of agreement was acceptable, supporting the use of antral size as a measure of gastric emptying of liquids in normal subjects.

**Figure 4:**
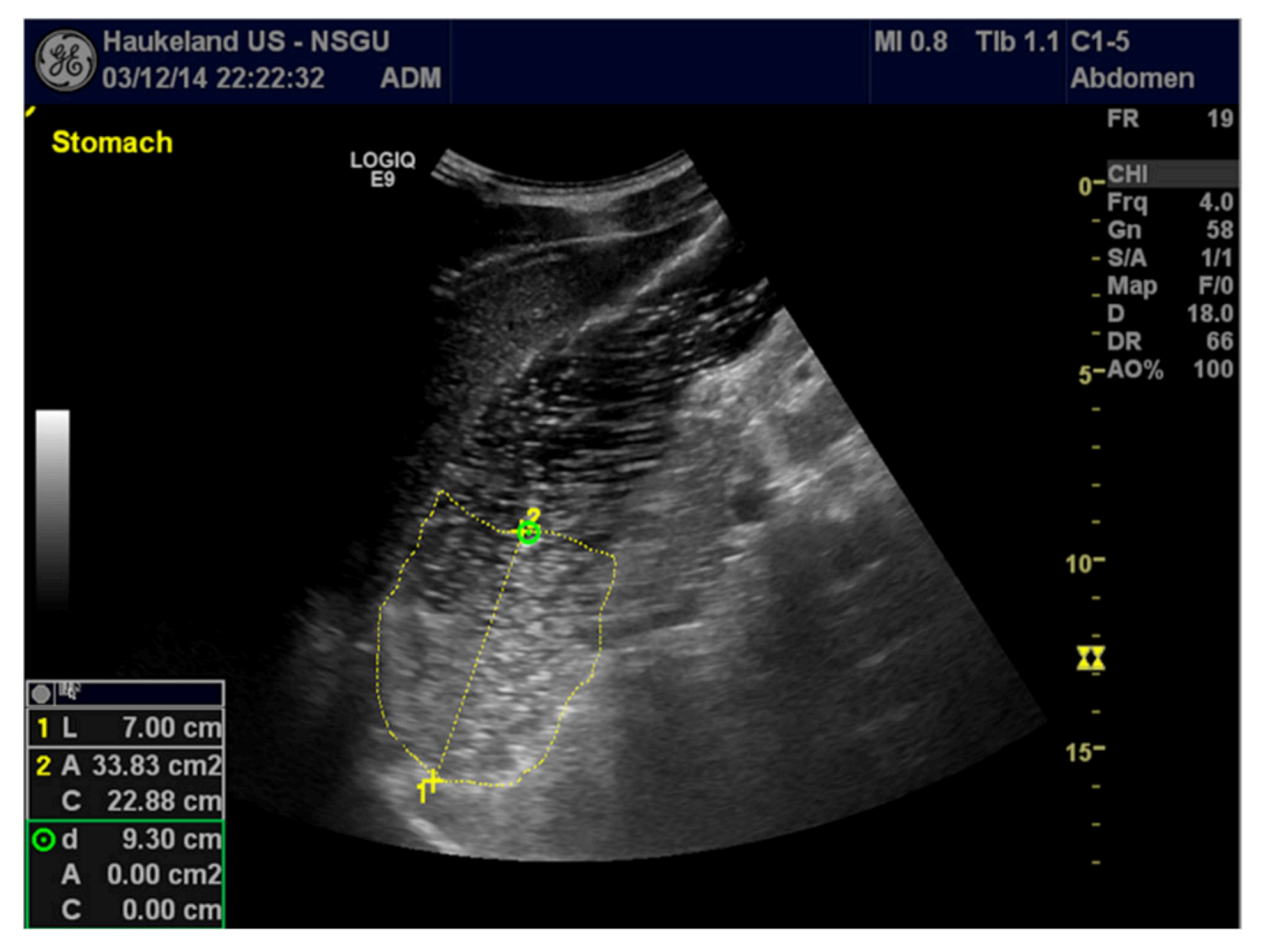
A 2D ultrasonographic image showing the proximal part of the stomach. The proximal stomach is typically measured in two dimensions, including a sagittal plane as shown here. The gastric area is measured using the left lobe of the liver, the left kidney, and the hemidiaphragm as anatomical landmarks.

**Figure 5:**
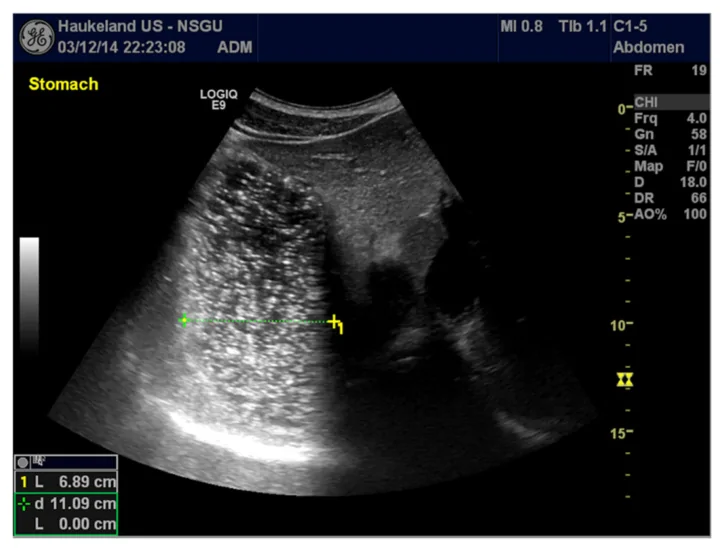
A 2D ultrasonographic image showing the proximal part of the stomach. The second measurement is obtained in an oblique frontal plane, in which the gastric diameter is measured using the hemidiaphragm as an anatomical landmark.

Several studies have explored alternative gastric regions for the assessment of gastric emptying, including the proximal stomach. Gilja et al. developed a new method to measure the size and volume of the proximal stomach using 2D ultrasound [29,30]. Images of the proximal stomach are illustrated in Figure 4 and Figure 5. By positioning the ultrasound probe in the epigastrium and tilting it cranially, it was possible to obtain 2D sections of the fluid-filled proximal stomach. In a later study using the same method [5], gastric emptying rates were evaluated in patients with type 1 and type 2 diabetes with and without gastroparesis, as well as in healthy controls, using ultrasound and compared with scintigraphy. Ultrasound parameters included measurements of the antral area, proximal diameter, and proximal area. The proximal diameter was obtained from a frontal oblique plane, whereas the proximal area was measured in a sagittal section along the epigastric midline, calculated as the area located 7 cm from the gastric apex. Measurement of the proximal stomach at 20 min postprandially correlated significantly with scintigraphic measurements at 2 h (r = 0.510, *p* = 0.001) and 4 h (r = 0.528, *p* = 0.001).

While 2D ultrasonography has been used to quantify antral wall motion, short-term patterns of transpyloric flow, and gastric emptying [20], it has some limitations. Gastric volume is usually estimated from measurements of antral cross-sectional area using the sum-of-cylinders method or mathematical algorithms that rely on geometric assumptions [20,43]. 3D ultrasound methods were developed to enable improved estimation of gastric volumes, thus enabling more precise assessment of gastric emptying and intragastric meal distribution [25,31].

In the study by Gentilcore et al. [20], healthy participants underwent simultaneous measurement of gastric emptying of high- and low-nutrient drinks with scintigraphy and a 3D ultrasonography method. Direct gastric volume measurements were assessed by placing the subject between a magnetic transmitter and the ultrasound transducer attached to a sensor allowing 3D positioning and orientation measurements (POM). The stomach was scanned by a continuous translational movement along its long axis, generating transverse sections of the entire stomach. The volume was calculated and expressed as a percentage of the original volume, and half-emptying time (T50) was also determined. There was no significant difference between the half-emptying time measured by 3D ultrasonography and by scintigraphy, including no difference in the overall emptying curves. Moreover, strong correlations between the two methods were observed for both the high-nutrient drink (r = 0.88, *p* = 0.007) and the low-nutrient drink (r = 0.92, *p* = 0.005). However, the study failed to demonstrate a superior correlation between 3D ultrasonography and scintigraphy compared with that observed between 2D ultrasonography and scintigraphy [17,20].

Similarly, Stevens et al. [43] conducted a study in patients with diabetes and gastroparesis, applying the same 3D ultrasound method to compare results with scintigraphy using a high-nutrient drink. Total gastric volume and overall gastric emptying time were assessed, in addition to regional volumes and emptying times of both the proximal and distal stomach. Conventional 2D ultrasound measurements were also performed, including assessment of the antral area and proximal area, allowing comparison between 2D and 3D ultrasound techniques as well as with scintigraphic findings. The 3D ultrasound method showed good agreement and concordance with scintigraphy and the 2D ultrasound measurements showed weaker correlations with scintigraphy and poorer agreement compared with the 3D POM method. The authors concluded that the 3D ultrasound method is a valid measure of gastric emptying in patients with gastroparesis.

Shen et al. [21] described an alternative 3D ultrasound technique, referred to as ultrasonic whole stomach cylinder measurements (UWSCM), and compared it with scintigraphy using a semisolid meal composed of a cellulose-based ultrasound contrast paste in healthy individuals. Total gastric volume was calculated by summing the volumes of the gastric body and the gastric antrum. These volumes were estimated using cylinder-based formulas derived from the longitudinal diameter of each segment and two to three perpendicular anterioposterior diameters, measured at intervals corresponding to one-quarter or one-third of the segment length. The total gastric volume was then plotted over time to determine the gastric half-emptying time. In addition to comparing this quasi-3D ultrasound method with scintigraphy, the authors also evaluated gastric emptying using conventional antral area and antral volume measurements. The findings demonstrated a highly significant correlation between whole-stomach cylinder volumes and scintigraphic measurements.

Several novel approaches for assessing gastric volume using 3D ultrasonography have been described [26,27]. Buisman et al. [26] evaluated a 3D ultrasound matrix technique and compared it with dynamic MRI. In this method, gastric volumes were obtained using a matrix transducer that provides an instantaneous volumetric field of approximately 90° × 90° when positioned over the stomach. Volume calculations were performed using dedicated software (QLAB quantification software; Philips Healthcare), which enabled image rendering and volumetric analysis. This technique allows for the assessment of total gastric volume, including intragastric air as well as liquid gastric volumes. In cases where full visualization of the stomach was challenging, a gel pad was used to improve the acoustic window. Unlike other 3D ultrasound methods, this approach does not require an external position and orientation measurement (POM) system or mechanical probe movement, as volume acquisition is achieved through a dense matrix phased-array transducer. The study demonstrated excellent agreement between matrix-based 3D ultrasonography and dynamic MRI in healthy adults. Strong correlations were observed for both total gastric volume and liquid gastric volume, with no significant differences in gastric emptying curves between the two modalities. Although this technique has not yet been applied in gastric emptying studies, it shows promise for future research.

#### 3.2.2. Advantages

Ultrasound is widely available, inexpensive, non-invasive, portable, and does not expose patients to ionizing radiation [13,19,28,71]. It is also safe, reproducible and may be repeated as often as required without any risk to the patient or the physician, as it can be used where radiation exposure is to be avoided [16,65]. This is particularly advantageous for patient groups where scintigraphy is not advisable, such as newborns [44], children and pregnant women [5,41,52].

**Figure 6:**
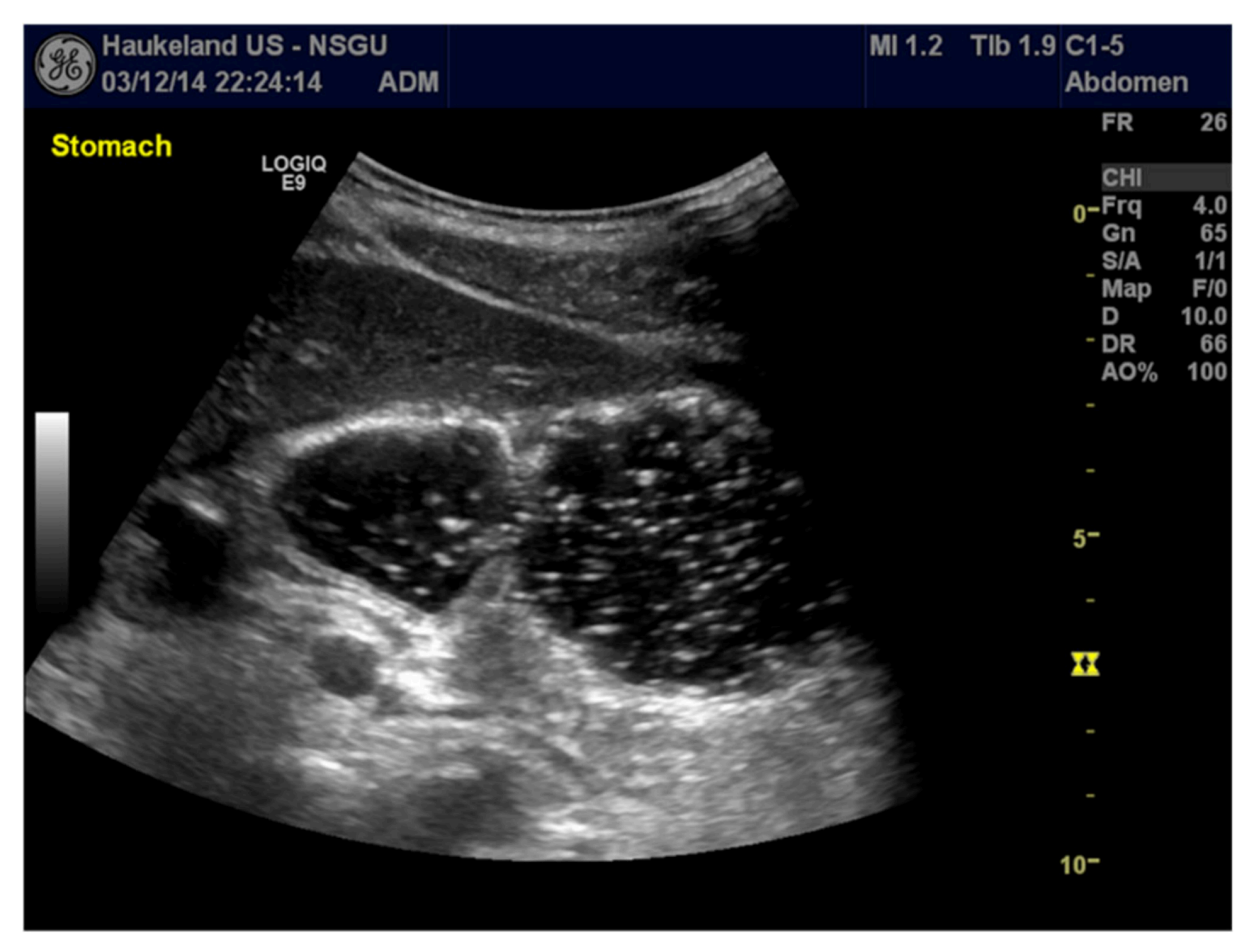
A 2D ultrasonographic image showing an antral contraction. Ultrasound is a real-time imaging modality with high temporal and spatial resolution, making it particularly well suited for assessing gastric motility.

Ultrasound can provide information, not only about gastric emptying, but also about gastroduodenal flow, contractility [28,69,70], gastric distension and accommodation [8,29,30], antral wall motility [13] and strain [32]. This allows dynamic imaging of peristaltic activity [41] including simultaneous estimation of gastric volume and antral contractions [51]. Figure 6 demonstrates an image of an antral contraction. By employing Doppler ultrasonography, it is possible to measure duodeno-gastric reflux and transpyloric flow of liquid meals [2,8]. It allows real-time assessment of the direction and characteristics of flow across the pylorus. A short gush of duodeno-gastric reflux normally occurs immediately before peristaltic closure of the pylorus. Episodes of gastric emptying can be identified as transpyloric flow with a mean velocity exceeding 10 cm/s and a duration of approximately 1 s. With this Doppler method, the timing of postprandial dyspeptic symptoms and transpyloric gastric contents can be measured with high temporal and spatial resolution and allows the relationship between transpyloric passage of gastric contents and postprandial symptoms to be investigated, as well as the effects of pharmacological interventions [8].

In addition, ultrasound is increasingly used by anesthesiologists as a point-of-care tool to assess preoperative/procedural gastric contents [5]. Assessment of aspiration risk is particularly important in patients with uncertain fasting status. This includes individuals who have not adhered to fasting guidelines, those with delayed gastric emptying, and those with an unreliable medical history because of language barriers or cognitive impairment. In these situations, gastric ultrasound may help guide perioperative decision-making and potentially prevent unnecessary surgical cancellations or rescheduling of elective procedures due to uncertainty regarding aspiration risk [72]. This issue has achieved recent prominence in relation to GLP-1 receptor agonists, which have been shown to increase residual gastric content despite appropriate fasting [73]. Bedside gastric ultrasound can be used to evaluate the nature and volume of gastric contents and thereby estimate aspiration risk [74]. An empty stomach is generally associated with a low risk of aspiration, whereas the presence of solid, particulate, or thick fluid contents indicates a substantially higher risk [72]. There is evidence that patients with type 2 diabetes, treated with GLP-1 receptor agonists, are at an increased risk of pulmonary aspiration following endoscopic procedures in patients receiving these medications [75,76], although current information is limited and inconsistent.

While ultrasound estimates of liquid half-emptying time are closely correlated with scintigraphic measurements [8,13], in addition, unlike scintigraphy, ultrasound is able to measure gastric secretions, as the volume that is measured represents the sum of the test meal and gastric secretions produced in response to the ingested food [16].

3D ultrasonography has been reported to be comparable in sensitivity to scintigraphy in evaluation of gastric emptying using liquid meals [20,42,43]. Importantly, 3D measurements also enable assessment of intragastric meal distribution and gastric volume without relying on assumptions about the geometric shape of the stomach [43]. Hence, 3D ultrasound allows precise volumetric assessment as well as direct measurement of both total and partial gastric volumes. Intragastric volumes can, therefore, be assessed more comprehensively, enabling measurement of total gastric volume, including fasting gastric volume, postprandial drink volume, and gastric secretions produced in response to the ingested meal, unlike scintigraphy [20]. The technique provides high spatial and temporal resolution, particularly compared with scintigraphy, as well as detailed anatomical visualization of the stomach.

#### 3.2.3. Disadvantages

Ultrasound measurements are operator-dependent and reliable results require a skilled examiner [2,13,58]. Furthermore, ultrasonographic assessment of gastric emptying is usually limited to liquids and semisolid meals [1,14,18]. Evaluation of solid gastric contents is technically more challenging, mainly due to excessive intragastric air, and is therefore subject to methodological limitations [24]. Although solid meals can adequately be visualized in the antrum, proximal gastric measurements following a solid meal may be more difficult to obtain because of gas entrapment in the corpus-fundus region of the stomach [5,19]. In addition, liquids with high fat or high protein content may also impair image quality using ultrasound [5]. While liquid emptying is generally easier to measure, solid emptying more closely reflects the physiological conditions encountered during normal meal consumption and may, therefore, be of greater clinical relevance [54]. Nevertheless, some studies have reported promising results in ultrasound estimation of gastric emptying using solid meals [19,41,65,70].

The use of ultrasound to assess gastric emptying assumes that a clear image of the stomach can be obtained. In addition to the challenges associated with visualizing solid meal components, accurate measurements may be limited by reduced visualization of the stomach in individuals with markedly increased subcutaneous or intra-abdominal fat. While some studies have excluded participants due to impaired visualization [5,16,49], others have reported that image quality was not significantly affected [20,43].

Good interobserver agreement has been reported for ultrasound measurements; however, considerable day-to-day variability has been observed in healthy individuals [77], but this is inconsistent [29]. This variability is thought to reflect physiological variations within the population rather than differences in measurement techniques and also applies to many of the other methods used to estimate gastric emptying. Consequently, large sample sizes are often required to demonstrate clinically meaningful changes in gastric emptying. In patients with gastroparesis, ultrasound may also have limitations in accurately assessing gastric emptying, as liquid emptying can, particularly that of low- or non-nutrient liquids, remain preserved even in advanced stages of the disease [8].

3D ultrasonography has some limitations. It is not as widely available as 2D ultrasonography and is primarily suited for assessing liquid gastric emptying, although particulate matter may be visualized in the fundus on scans [20]. Challenges include that the posterior wall of the stomach may be difficult to identify after the ingestion of solids [42]. Measurement of gastric emptying using solids may be feasible with 3D ultrasonography, but further studies are required to validate the technique [8]. As discussed, gastric emptying of solids may be more frequently delayed than the emptying of low-nutrient liquids in patients with gastric motility disorders, highlighting the importance of measuring solid gastric emptying [42]. Image quality may also be affected by intragastric air, particularly in the fundus, which can obscure the gastric outline; however, some studies report that this did not significantly affect the examination [20]. In addition, 3D ultrasonographic methods that use magnetic transmitters may be susceptible to magnetic interference, which may lead to spatial distortion of the images [20]. Measurements require experienced operators and appropriate technical expertise [42,43]. While numerous studies have investigated ultrasound assessment of gastric emptying, the available evidence remains heterogeneous with respect to study populations, meal composition, ultrasound techniques, and outcome measures. The majority of validation studies have been conducted in small cohorts of healthy individuals, whereas evidence relating to solid meals and clinical populations remains limited.

#### 3.2.4. Clinical Applications

Ultrasonography of the stomach is preferable in children and in pregnancy—and may be indicated in the evaluation of patients with dysmotility-related dyspepsia, as well as in patients with delayed gastric emptying, associated with both dyspeptic and non-dyspeptic conditions secondary to chronic disease. Such conditions include diabetes, systemic sclerosis, and myotonic dystrophy [58].

In patients with diabetes, delayed gastric emptying may result from several disturbances in gastroduodenal motor function, including reduced antral contractions, impaired coordination between the antrum and duodenum, increased pyloric contractions, and impaired proximal gastric relaxation [43]. Autonomic dysfunction, particularly impaired vagal function, is considered an important contributor to these abnormalities [5], while alterations in enteric neural function may further impair effective gastric peristalsis [63]. These disturbances can result in abnormal intragastric meal distribution and delayed gastric emptying. Ultrasonography may provide complementary information by allowing real-time assessment of gastric volume, antral cross-sectional area, and gastric motility. Changes in antral area over time can be used to estimate gastric emptying, while 3D ultrasonography enables more direct assessment of gastric volume and intragastric meal distribution. In patients with diabetic gastroparesis, 3D ultrasonographic and scintigraphic measurements of gastric emptying are significantly related, with good agreement, supporting the potential use of ultrasound as a non-invasive method for assessing gastric emptying in this population [43].

In functional dyspepsia patients, impaired gastric accommodation, antral hypomotility and delayed gastric emptying have been reported as major pathogenetic factors [2]. Ultrasonography has been reported to be a simple, non-invasive modality for assessment of gastric emptying and antral motility in either a liquid or solid meal [2].

In patients who have undergone surgery of the stomach, assessment of gastric emptying may add valuable information about residual function of the stomach. In addition, ultrasonography can be used to monitor the effects of pharmacological and non-pharmacological interventions that may influence gastric motility [16,58], and in the assessment of newborns with suspected hypertrophic pyloric stenosis [58].

## 4. Discussion

This review has considered two alternative methods to assess gastric emptying, with the aim of comparing their advantages and disadvantages. The literature review revealed that scintigraphy is a quantitative, easy-to-perform, and widely available method that involves a relatively low radiation burden. In addition, it provides information on physiological aspects of gastric emptying. It enables assessment of antral contractions and intragastric meal distribution. However, the method is limited by a lack of standardization regarding meal composition, patient positioning, timing of image acquisition, and the availability of appropriate normal ranges. In addition, the procedure is time-consuming, cannot measure salivary or gastric secretions or differentiate between the layers of the gastric wall, and measurements may be inaccurately assessed due to overlap of the small intestine.

For a method to be suitable for routine clinical monitoring, it must be safe. While scintigraphy involves a relatively low radiation dose, it still exposes patients to ionizing radiation, creating a small but present risk of cumulative exposure when repeated examinations are performed [17]. This consideration is particularly important in pediatric populations, where delayed gastric emptying may be part of a broader motility disorder in children and lead to symptoms such as abdominal pain, bloating, nausea, vomiting, and failure to thrive [78]. This highlights the importance of having diagnostic methods that are safe and suitable for use in this patient group. In contrast to scintigraphy, both 2D and 3D ultrasound methods are safe for use in children and have been widely applied [79,80]. In addition to safety, the method should also be cost-effective. The use of expensive equipment, the time required to complete an examination for a single patient, and the need for trained personnel all contribute to increased costs. These factors limit the use of scintigraphy as a routine clinical monitoring tool.

Ultrasound is a patient-friendly, inexpensive, and widely available imaging modality that does not involve exposure to ionizing radiation. As well as assessing gastric emptying, it also enables evaluation of gastric contractility, proximal gastric accommodation, gastrointestinal wall strain, and transpyloric flow with high spatial and temporal resolution. However, the majority of validation studies are small and heterogeneous in relation to meal composition, study population, ultrasound techniques and outcome measures. Furthermore, the majority have been conducted in single centers and many of the studies have included only healthy volunteers, limiting the generalizability of the findings to broader clinical populations. Standardized examination protocols and widely accepted reference ranges are lacking [53], compromising comparisons across studies. Larger studies are, therefore, required to establish appropriate normal ranges for specific test meals [8,57]. Furthermore, the evidence is strongest with liquid meals and selected research settings, while evidence for solid meals and routine clinical diagnoses remains limited, including both 2D and 3D ultrasonography. Available evidence suggests that 3D ultrasonography is an acceptable, non-invasive method for assessing gastric emptying in healthy individuals and patients with gastroparesis [20,43]. 3D ultrasonography generally requires less costly equipment than scintigraphy [17]. Because it enables simultaneous assessment of the proximal and distal stomach, 3D ultrasonography allows assessment of gastric accommodation while quantifying gastric emptying [43]. 3D ultrasonography may also provide a more comprehensive assessment of gastric emptying than 2D ultrasonography, particularly through direct measurement of gastric volume. As previous studies have demonstrated good agreement with scintigraphy using liquid meals [20,43], future research should focus on further validation with solid meals as well as establishing an acceptable normal range of gastric emptying [20].

A limitation of this review is that only two methods to measure gastric emptying were assessed. MRI, stable isotope breath tests and the paracetamol absorption test have also been utilized to assess gastric emptying [1,55], albeit the latter has significant limitations and should be avoided [81].

In conclusion, both scintigraphy and ultrasound are valuable methods for the assessment of gastric emptying, each with distinct strengths and limitations. Scintigraphy remains the clinical reference standard due to its wide acceptance and ability to provide quantitative and physiologically relevant measurements, especially for assessment of gastric emptying of solids. However, its clinical applicability is limited by radiation exposure, lack of standardization, and cost. Both 2D and 3D ultrasonography have demonstrated strong correlations and good agreement with scintigraphy in the assessment of gastric emptying. 3D ultrasonography shows promise as a safe, non-invasive method for assessing gastric emptying, although further larger clinical studies are required before routine implementation. The majority of validation studies of 2D and 3D ultrasonography are small and heterogeneous. The most consistent evidence is available for liquid meals and selected research settings, whereas evidence relating to solid meals and routine clinical diagnosis remains limited and standardized protocols and reference ranges are lacking. Until ultrasound protocols are further refined and validated, particularly for solid-meal gastric emptying assessment, scintigraphy remains the gold standard. Further efforts are required to promote harmonization and consistent implementation of established consensus recommendations for scintigraphic measurement of gastric emptying across centers. Additional research is also required to validate ultrasonographic measurements to define their potential role in clinical practice.

## Figures and Tables

**Table 1:** Summary of studies comparing scintigraphic and ultrasonographic measurements of gastric emptying, including the reference and year of publication, the number and characteristics of the study subjects, as well as details on the meal type, and ultrasound and scintigraphic method used.

Reference	*N*	Population	Meal Type	Ultrasound Method	Scintigraphy Method
Holt et al., 1986 [16]	14	Healthy subjects, duodenal and gastric ulcer and diabetic patients	Liquid, 300 mL beef extract	Measured total gastric volume from cross -sectional measurements	^99m^Tc-sulfur colloid Measured anterior aspect of abdomen
Tympner et al., 1986 [45]	12	Diabetes mellitus patients	Liquid, 200 mL water + sachet of antacid 50 mL	Measured the proximal area and viewed when echoic liquid had passed.	^99m^Tc-methylene Diphosphonate (MDP) Measured anterior aspect of the stomach
Sauer et al., 1988 [49]	12	Suspected diabetes mellitus and alcohol-related disease	Liquid, 300 mL orange juice	Measured the cross-sectional antral area in 2 different sections	^99m^Tc Measured anterior–posterior aspect of the stomach
Marzio et al., 1989 [18]	29	Healthy subjects	Liquid, 500 mL saline solution or skimmed milk	Measured anterior–posterior and latero-lateral gastric measurements of the antrum	^99m^Tc-DTPA Measured anterior–posterior aspect of the stomach
Wedmann et al., 1990 [48]	35	Healthy controls and gastroparetic patients	Liquid, 300 mL of water solution and Biloptin	Measured the antral cross-sectional area	^99m^Tc-sulfur colloid Measured anterior–posterior aspect of abdomen
Dapoigny et al., 1991 [46]	26	Healthy controls and patients with GI symptoms or transit bowel issues (IBS or FD)	Liquid and solid, chicken liver and orange juice (2000 kj)	Measured the antral cross-sectional area	^111^In-DTPA in orange juice and ^99m^Tc in chicken liver. Measured anterior–posterior aspect of the stomach
Takaoka et al., 1993 [40]	7	Healthy subjects	Liquid, 400 mL ensure liquid (400 kcal)	Measured cross-sectional areas in antrum and gastric body	^99m^Tc-DTPA Measured anterior aspect of the stomach
Duan et al., 1993 [3]	24	Non-ulcer dyspepsia patients	Mixed solids, Chinese meal (270 kcal)	Measured cross-sectional area and volume of the antrum using longitudinal and anterior–posterior diameters.	^99m^Tc-sulfur colloid
Prassler et al., 1996 [50]	63	Healthy controls and diabetes mellitus patients	Solid, 300 mL oatmeal or 125 g potato pancakes	Measured the antral cross-sectional area	Measured anterior–posterior aspect of the stomach
Okamoto et al., 1997 [51]	20	Healthy men	Liquid, 400 mL consommé (13.1 kcal) Solid, egg bowl (520 kcal)	Measured antral cross-sectional area	^99m^Tc
Benini et al., 1999 [19]	19	Healthy subjects, gastroesophageal reflux and dysmotility-like dyspepsia	Solid, Bolognese meal (800 kcal)	Measured antral cross -sectional area	^99m^Tc-colloid Measured anterior aspect of abdomen
Gomes et al., 2003 [52]	10	Babies with vomiting and failure to thrive	Liquid, 25 mL milk/kg	Measured antral orthogonal cross-sectional area, reported filling index	^99m^Tc-phytate (200 uCi) Measured anterior–posterior aspect of stomach
Darwiche et al., 2003 [41]	11	Insulin-dependent diabetes mellitus patients	Semisolid, 300 g rice pudding and egg (1764 kJ)	Measured antral cross -sectional area	^99m^Tc medronate disodium Measured anterior–posterior aspect of abdomen
Gentilcore et al., 2006 [20]	10	Healthy subjects	Liquid, 500 mL beef broth (20 kcal) or 300 mL dextrose (314 kcal)	3D ultrasound with positioning and orientation measurement (POM)	^99m^Tc-sulfur colloid Measured anterior–posterior aspect of abdomen
Engelmann et al., 2010 [44]	26	Children with neurologically impaired/unimpaired who underwent laparoscopic anterior 270° fundoplication	Liquid, 10 mL water	Measured antral diameters	^99m^Tc-DTPA
Stevens et al., 2011 [43]	10	Diabetes mellitus patients with gastroparesis	Liquid, 75 g dextrose in 300 mL water	3D ultrasound with positioning and orientation measurement (POM)	^99m^Tc-sulfur colloid Measured posterior aspect of abdomen
Shen et al., 2014 [21]	128	Healthy subjects and type 2 diabetes patients	Semisolid, 400 mL ultrasonic carrier agent	Measured cross-sectional antral area, antral volume, gastric body volume and whole stomach volume	^99m^Tc
Liu et al., 2018 [42]	10	Healthy subjects	Semisolid, 350 mL cellulose-based paste	3D ultrasound, volume measurements calculated automatically from multiple 2D images	^99m^Tc-sulfur colloid Measured anterior aspect of abdomen
Steinsvik et al., 2022 [5]	88	Healthy controls, patients with diabetes and symptoms of gastroparesis	Liquid, 500 mL meat soup (84 kJ) (Ultrasound) Solid, 350 kcal egg and nutrient bar	Measured antral cross-sectional area, proximal area and proximal diameter	^99m^Tc-nano colloid Measured anterior–posterior aspect of abdomen
Wang et al., 2026 [38]	113	Gastroparesis patients	Solid, 120 g liquid egg white, 2 slices of bread and jam (scintigraphy) Liquid, 48 g of commercial gastric ultrasound powder dissolved in water (ultrasound)	Antral cross-sectional area, gastric volume and antral motivity index	^99m^Tc-nano colloid Anterior–posterior image of the upper abdomen

**Table 2:** Advantages and disadvantages of scintigraphy and ultrasound for assessing gastric emptying. This Table summarizes the key features of the techniques.

Method	Scintigraphy	Ultrasonography
Functional information	Gastric emptying, intragastric meal distribution, antral motor function	Gastric emptying, accommodation, antral contractility, transpyloric flow, wall strain
Anatomical information	Limited	Yes
Examination duration	2–4 h	30–60 min
Region addressed	Entire stomach	2D: primarily antrum; 3D: entire stomach
Radiation exposure	Yes	No
Widely available	Moderate (requires nuclear medicine facilities)	Yes
Cost	Relatively high	Relatively low
Operator dependent	Low	High
Technical expertise and training requirements	Standardized acquisition; specialist nuclear medicine staff	Requires experienced operator and standardized technique
Portability/bedside use	No	Yes
Suitable for children	Limited by radiation exposure	Yes
Suitability during pregnancy	Limited by radiation exposure	Yes
Temporal resolution	Intermittent image acquisition	Real-time imaging with high temporal resolution
Spatial resolution	Low	High
Repeatability	Limited by radiation exposure	Yes
Reproducibility	High with standardized protocols	Moderate to high
Standardized protocols	Consensus recommendations available, not universally adopted	Limited standardization
Reference ranges	Available for standardized protocols	Limited standardization
Assessment of liquid meals	Good	Good
Assessment of solid meals	Good	Limited
Assessment of semisolid meals	Good	Limited
Limitations related to obesity	Minimal	May reduce image quality
Limitations related to intragastric gas	Minimal	May impair visualization
Limitations due to intestinal overlap	May interfere with quantification	May obscure gastric structures

**Figure A1:**
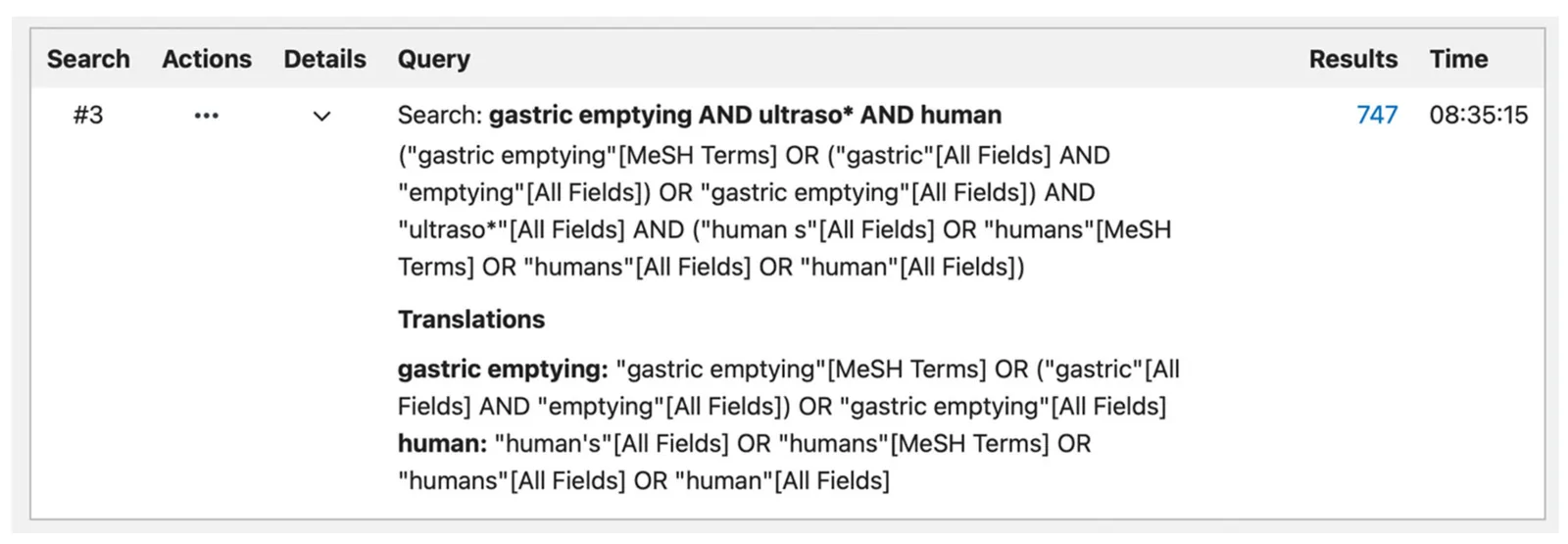
Initial searches conducted in PubMed on 11 December 2024, using the terms ‘gastric emptying,’ ‘ultraso,’ and ‘human,’ yielded 747 articles.

**Figure A2:**
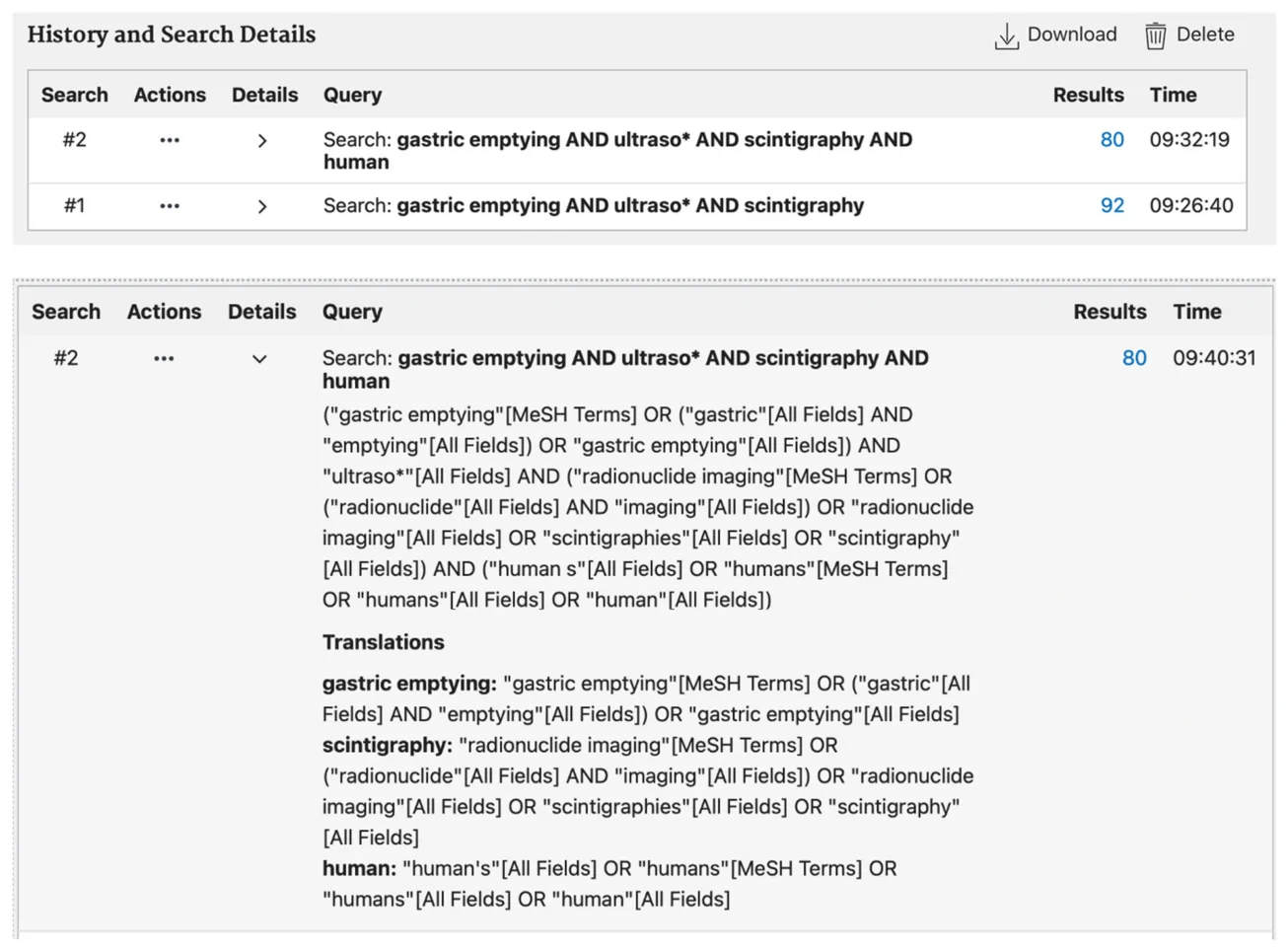
The finalized PubMed search conducted on 12 December 2024, using the keywords ‘gastric emptying’, ‘ultraso’, ‘scintigraphy’, and ‘human’, yielded 80 articles.

**Figure A3:**
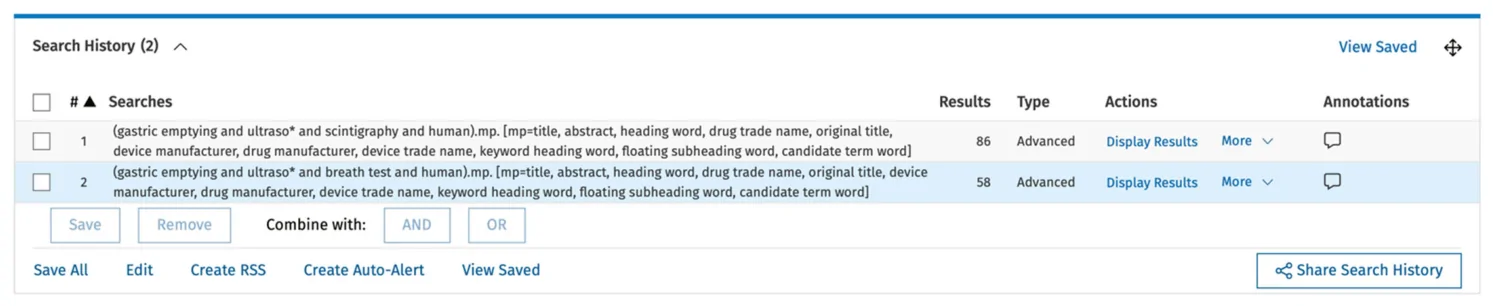
An additional search conducted in Embase on the 22nd of December 2024 completed the initial search strategy. The search used the same keywords as those applied in PubMed and yielded 86 results. After comparison of titles with the PubMed search, 28 additional articles were identified. In total, 108 articles were assessed and screened. An additional search was conducted on the same day using the keywords ‘gastric emptying’, ‘ultraso’, ‘breath test’, and ‘human’, yielding 58 results; however, to limit the number of articles, the articles identified through this search were excluded.

**Figure A4:**
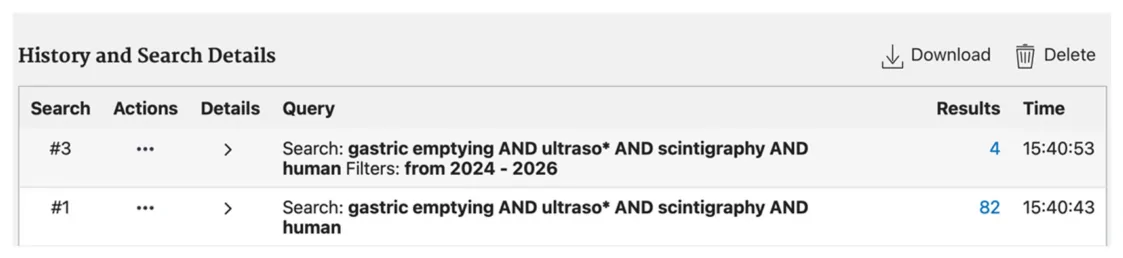
PubMed search conducted in August 2026, which identified two additional articles to the initial search, of which one met the eligibility criteria for inclusion in the review.

**Figure A5:**
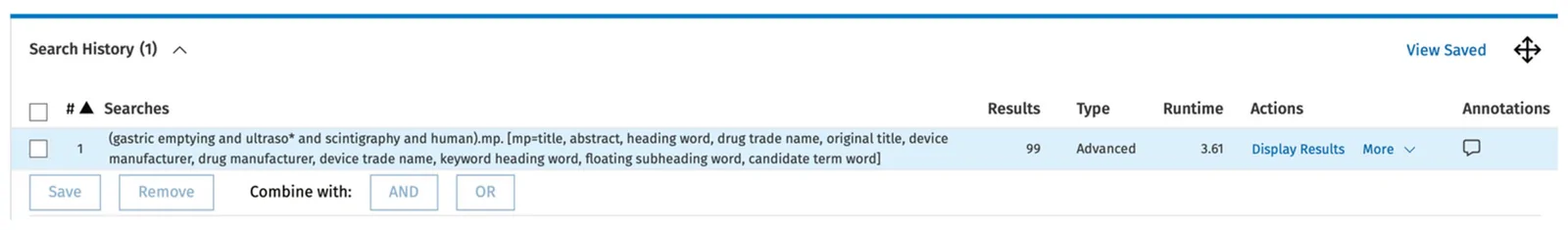
Embase search conducted in August 2026, which identified 13 articles in addition to the initial search, of which one met the eligibility criteria described in the Methods Section. Following the updated search in August 2026, a total of 46 articles were included in the review.

## Data Availability

No new data were created or analyzed in this study. Data sharing is not applicable to this article.
